# The wisdom of crowds and the repurposing of artesunate as an anticancer drug

**DOI:** 10.3332/ecancer.2015.ed50

**Published:** 2015-10-13

**Authors:** Yolanda Augustin, Sanjeev Krishna, Devinder Kumar, Pan Pantziarka

**Affiliations:** 1 Molecular and Medical Parasitology Group, St George’s, University of London, Cranmer Terrace, SW17 0RE, UK; 2 Department of Surgery, St. George’s, University of London, Cranmer Terrace, SW17 0RE, UK; 3 Anticancer Fund, Brussels, 1853 Strombeek-Bever, Belgium; 4 The George Pantziarka TP53 Trust, London KT1 2JP, UK

## Abstract

Artesunate, a semi-synthetic and water-soluble artemisinin-derivative used as an anti-malarial agent, has attracted the attention of cancer researchers due to a broad range of anti-cancer activity including anti-angiogenic, immunomodulatory and treatment-sensitisation effects. In addition to pre-clinical evidence in a range of cancers, a recently completed randomised blinded trial in colorectal cancer has provided a positive signal for further clinical investigation. Used perioperatively artesunate appears to reduce the rate of disease recurrence - and the Neo-Art trial, a larger Phase II RCT, is seeking to confirm this positive effect. However, artesunate is a generic medication, and as with other trials of repurposed drugs, the Neo-Art trial does not have commercial sponsorship. In an innovative move, the trial is seeking funds directly from members of the public via a crowd-funding strategy that may have resonance beyond this single trial.

The artemisinins, a group of drugs derived from *Artemisia annua L* (sweet wormwood), comprise an interesting set of small molecules with diverse activities in a broad range of diseases. With a long history of use as traditional medicines, the artemisinins emerged as potent and widely used anti-malarial drugs following a sustained program of development initiated by the Chinese government in the 1970s and 80s [1, 2]. A number of these compounds, in particular the semi-synthetic and water-soluble artemisinin-derivative artesunate, soon attracted the interest of researchers looking for new anticancer agents [3, 4]. To date multiple mechanisms of action have been posited to explain the *in vitro* and *in vivo* evidence of anticancer activity, including anti-angiogenesis, reversal of multi-drug resistance, ROS-induced DNA damage, immunostimulation, improved radiosensitivity and others.

While much of the cancer-related research on artesunate has had a pre-clinical focus, some clinical work has also been initiated. Notable examples include a small open-label trial in advanced cervical cancer [5], a pharmacokinetic study in metastatic breast cancer [6], and an on-going Phase I dose escalation trial in hepatocellular carcinoma (NCT02304289). Recently a randomised controlled trial of oral artesunate in colorectal was completed at St George’s Hospital in the UK [7]. Patients awaiting curative resection of biopsy-confirmed colorectal cancer were assigned oral artesunate (n = 12), 200 mg per day, or placebo (n = 11) for 14 days prior to resection. The primary outcomes were biochemical, with measures of apoptosis the primary metric. However, a higher than expected level of apoptotic staining in the placebo group precluded detection of an artesunate effect on apoptosis, although secondary analyses suggested an anti-proliferative effect. Furthermore the treatment was well tolerated and toxicity was manageable. More interesting is that during a median follow-up period of 42 months a single patient in the artesunate group developed recurrent disease compared to six patients in the placebo group. We may conclude, therefore, that the trial provides a positive signal for further clinical investigation.

The Neo-ART trial is a Phase II randomised, double-blind, placebo-controlled trial of neo-adjuvant artesunate in patients (n = 140) with Stage II/III colorectal cancer treated with curative surgery. As with the preceding trial at St George’s, patients will be treated with oral artesunate, at a dose of 200 mg per day, for 14 days prior to surgical resection. The primary outcome will be recurrence free survival at two years, with overall survival and disease-specific survival included in the secondary outcomes.

There are a number of aspects of this trial which invite additional comment.

The first is that as with the Phase I trial, this is focused on a relatively short-term intervention during the peri-operative period. As is well known, the majority of cancer-related fatalities are due to metastatic disease and there is now an increased attention on the peri-operative period as providing a window of opportunity to address this major clinical issue [8, 9]. Artesunate is one of a number of agents which may have substantial impact on the reduction of post-surgical distant metastases, others include aspirin [10], ketorolac [11] and cimetidine [12]. The issue of high post-surgical recurrence rates is not unique to some types of colorectal cancer – it is a significant issue in breast, upper GI, NSCLC, osteosarcoma and other malignancies. Artesunate, then, may have the potential for a significantly positive effect in a number of other cancers in addition to colorectal disease.

Secondly, it is noteworthy that many of the drugs which have the potential to be used peri-operatively to reduce the recurrence rate are repurposed drugs. Drug repurposing as a strategy is of increasing interest in the oncology world [13, 14]. Artesunate, included in the WHO List of Essential Medicines for the treatment of malaria, is a generic drug with widespread use across low and middle income countries (LMIC). The potential benefit of low cost, low toxicity and generally available drugs such as artesunate are immense in both economic and social terms if they can be shown to be clinically effective in cancer. In contrast to the latest generation of targeted agents these are drugs which are both affordable and easy to administer – particularly if the intervention is of relatively short duration.

Finally, the Neo-ART trial also illustrates one of the obstacles that stands in the way of more widespread clinical investigation of repurposed drugs – the lack of a commercial sponsor. Paradoxically it is one of the benefits of repurposed drugs – relatively low-cost – which is also something of an impediment. A lack of financial incentives related to generic costs leaves us with a class of drugs which have been termed ‘financial orphans’ [15]. Without the prospect of a positive return on investment commercial sponsorship of clinical trials using generic drugs does not happen. In this scenario investigators need to adopt innovative approaches to solve the problem.

In the case of the Neo-ART trial the investigators have adopted a plan to ‘crowdfund’ the trial – that is they are seeking financial input from members of the public, small charities and other non-commercial sponsors. It is an approach that potentially engages patients and public to a greater degree than the traditional commercially-sponsored clinical trial. This is an experimental approach that may reverberate beyond this single trial and, if successful, signal the start of a new relationship between clinical researchers and the public they ultimately serve. This model would empower patients and communities to help drive research in areas important to them, and also enable clinicians and scientists to conduct research studies that are vital but for which they may struggle to obtain the funds required through conventional funding streams.

However, the ultimate aim of Neo-ART is to prove the clinical efficacy of artesunate in colorectal cancer not to pilot new models of trial-financing. Should the trial prove successful, the question remains as to how to move the drug into the clinic. Artesunate will need to be licensed for a new indication in cancer – for the moment this is not a question to which we have an answer.

Learn more about the project here: https://www.futsci.com/project/antimalarial-cancer.
